# Editorial: Micronutrient metabolism: molecular pathways and pharmacology

**DOI:** 10.3389/fnut.2024.1490425

**Published:** 2024-10-29

**Authors:** Paraskevi Detopoulou, Hamed Haghi-Aminjan, Mahban Rahimifard

**Affiliations:** ^1^Department of Nutritional Science and Dietetics, University of the Peloponnese, Kalamata, Greece; ^2^Pharmaceutical Sciences Research Center, Ardabil University of Medical Sciences, Ardabil, Iran; ^3^Toxicology and Diseases Group (TDG), Pharmaceutical Sciences Research Center (PSRC), Tehran University of Medical Sciences (TUMS), Tehran, Iran

**Keywords:** micronutrient, mineral, molecular pathways, pharmacology, vitamin

Micronutrients, in the context of a healthy diet, are vital to humans in small amounts. Inadequate intake, age, gene polymorphisms, disease, dysbiosis, and drug-nutrient interactions may influence their status and metabolic fate. In turn, suboptimal, deficient status or toxicity of micronutrients predispose to pathological conditions (1). Animal models are useful tools in the research mechanisms of micronutrients since they provide controlled conditions, while population-based studies help to clarify their role in real-life conditions. The present Research Topic on the pharmacology and molecular pathways of micronutrients provides fresh insights from animal models and human studies regarding several mechanistic aspects of micronutrients in health and disease.

A special focus is given to B-vitamins. Yu et al. demonstrated that B vitamin deficiency may affect the reproductive status of females. More particularly, the absence of vitamins B1, B3, B5, B6, or B7 significantly decreased the lifespan of wild-type *Drosophila* females, but had little effect on the lifespan of mutant females. Interestingly, vitamin B deficiency had an additional effect on lipid metabolism, highlighting the complexity of nutrient interactions. Of note, some previous reports have also pointed to an effect of thiamin and niacin on lipid metabolism (2, 3). Yang et al. have published a systematic review and meta-analysis clarifying the role of folic acid in primary stroke prevention. The meta-analysis suggested that high folic acid intake from the diet was associated with a decrease in stroke occurrence (RR: 0.83; 95% CI: 0.73–0.94), especially in regions without grain folate fortification. Similarly, supplements of folic acid had a protective role against stroke only in regions without grain fortification (RR: 0.78; 95% CI: 0.68–0.89). This analysis improved the current knowledge and facilitated clinical decision-making and public health strategies.

Furthermore, micronutrients such as magnesium, calcium, selenium, and zinc play a crucial role in human health by supporting various bodily functions, including immune response, bone health, and metabolic processes, highlighting the need for a balanced diet to ensure adequate intake of these essential nutrients (4, 5). Wu et al., in a study of 7,726 participants between 1999 and 2004 in the National Health and Nutrition Examination Survey (NHANES), explored the link between dietary calcium and magnesium intake and peripheral neuropathy. Using a multifactor logistic regression model and a restricted cubic spline plot, the research discovered a U-shaped non-linear relationship, revealing that both high and low levels of calcium and magnesium intake are associated with an increased risk of developing peripheral neuropathy. Another micronutrient that is very important in the diet is selenium. Hailu et al. conducted a study in Ethiopia on the relationship between the amount of selenium in the soil and its presence in the bodies of nursing mothers and their babies. In some studied areas in Ethiopia, we have seen selenium deficiency in breast milk and urine samples, and this has a significant relationship with the amount of selenium in the soil of the region. Considering the importance of selenium and its function, especially strengthening the immune system and reducing oxidative stress in the body (6), there is a need to implement targeted agricultural interventions to increase the concentration of this valuable substance in the daily food of these people.

On the other hand, the presence of vitamins and minerals in the body not only modulates metabolic processes and signaling pathways but also affects significantly the composition and function of the body's microbiome. In the study conducted by Zhang et al., it has been demonstrated that there is an inverse relationship between the occurrence of seropositivity for Helicobacter pylori and dietary zinc consumption, suggesting that higher zinc intake could be associated with lower rates of infection. Additionally, Sempértegui et al. identified an inverse relationship between gastric inflammation caused by Helicobacter pylori and the levels of zinc present in the gastric mucosa (7).

According to the studies, it is very important to follow a healthy diet to prevent many diseases. In another study of the present Research Topic, Chu et al. showed that hypocarnitinemia impacts seizure control in patients with refractory epilepsy following the modified Atkins diet. Last but not least, the associations of dietary patterns rich in antioxidants and/or particular micronutrients with body composition variables are also discussed in the present Research Topic. More particularly, the dietary antioxidant capacity was firstly associated with phase angle in the work of Detopoulou et al. Phase angle derives from bioelectrical impedance analysis raw data and relates to cellular health and membrane integrity (8). Other studies have shown a potential relationship of phase angle with protein and/or Mediterranean diet adherence (9, 10). The potential modulation of phase angle by antioxidants is of outmost importance since it serves as a prognostic factor in several diseases, including cancer and sarcopenia (11). In addition, the dietary antioxidant capacity was inversely associated with platelet activating factor (PAF), corroborating the relation of PAF with dietary factors (12, 13). The study of Liu et al. revealed that dietary patterns rich in antioxidants or other nutrients (B-vitamins and Fe, Zn, and Se or vitamins A, D, B12, and Ca) were inversely related to sarcopenia in US adults from the National Health and Nutrition Examination Survey (NHANES) 2011–2014. As previously suggested, antioxidants maintain redox status and can combat sarcopenia-related oxidative stress by reducing inflammatory mediators and muscle proteolysis (14). In this context, the present results suggest that micronutrient-rich dietary patterns may constitute a useful strategy for preserving muscle mass and Fighting sarcopenia.

Overall, the results of published studies in this Research Topic highlight several mechanisms and/or actions of minerals, vitamins, and micronutrients-rich dietary patterns in health and disease. They revealed associations between diet and sarcopenia, fertility, H. pylori infection, and stroke, underscoring the importance of lifestyle modifications in disease management and prevention. We anticipate that this Research Topic will serve as a guide for future scientists to further investigate the nature of nutrient interactions and the role of micronutrients in disease prevention.
